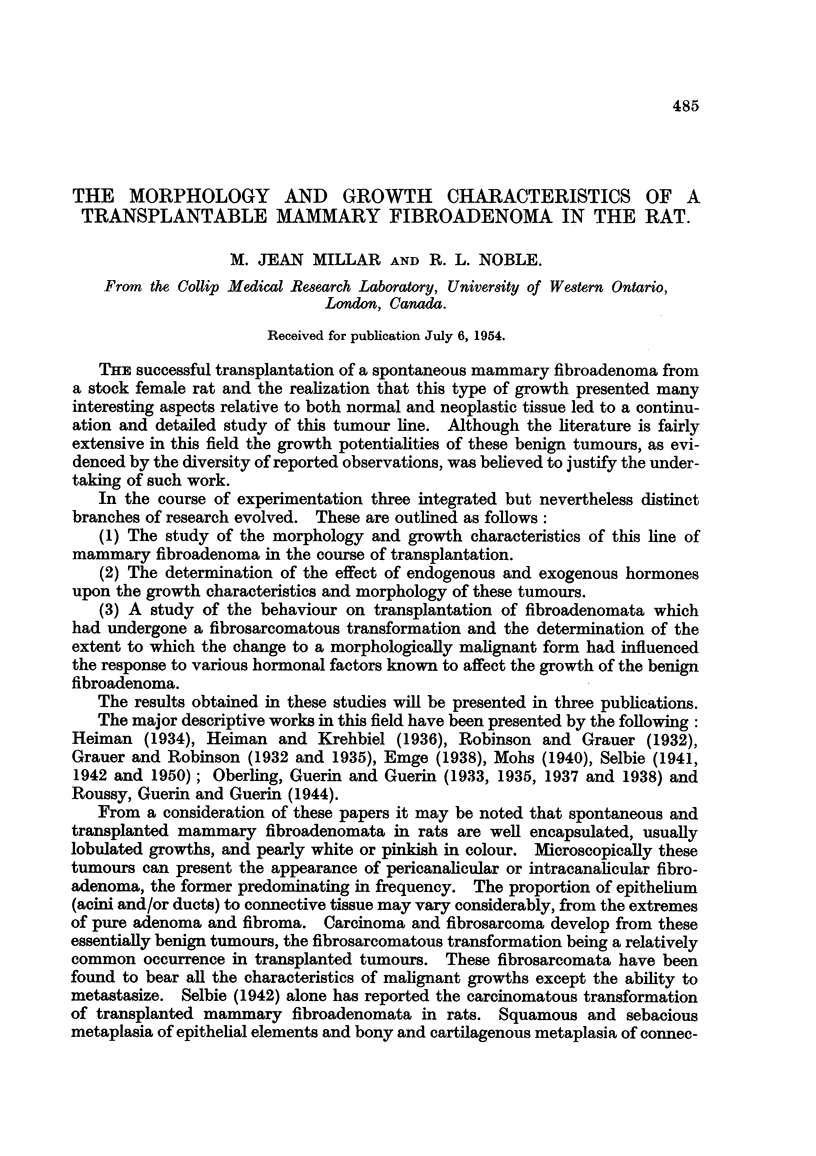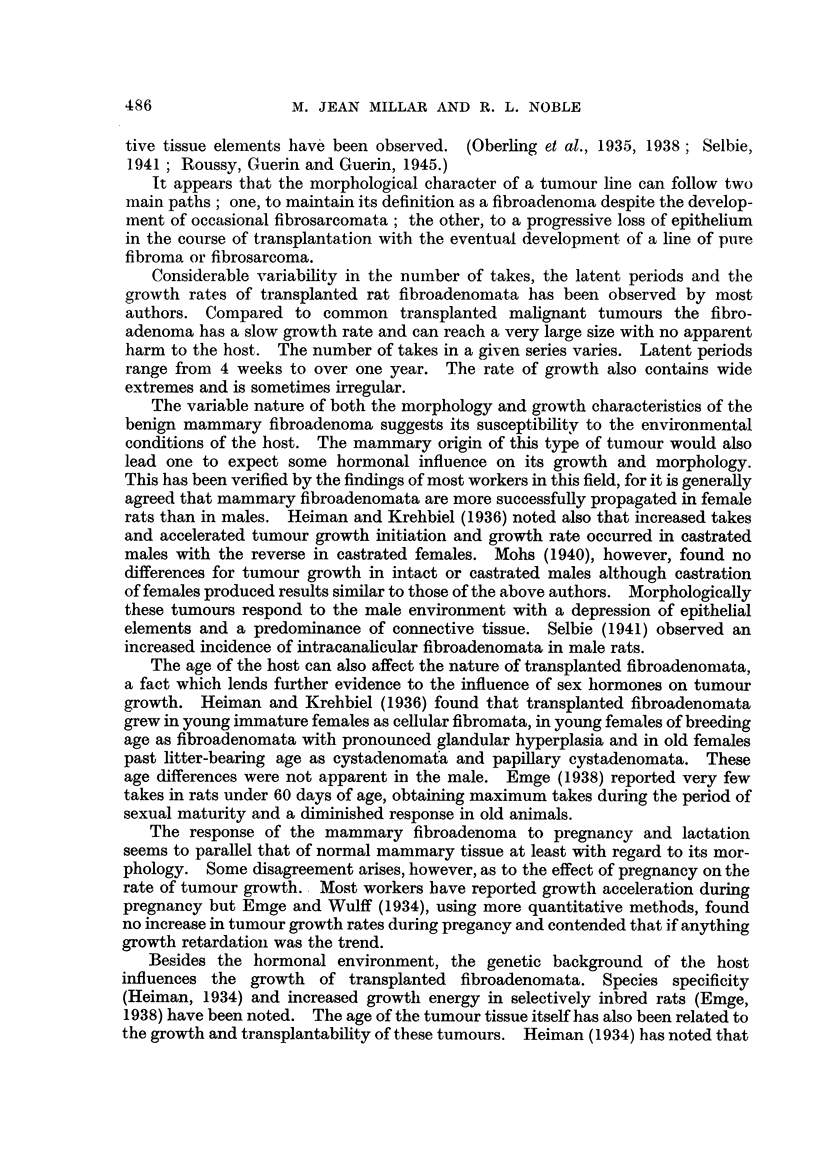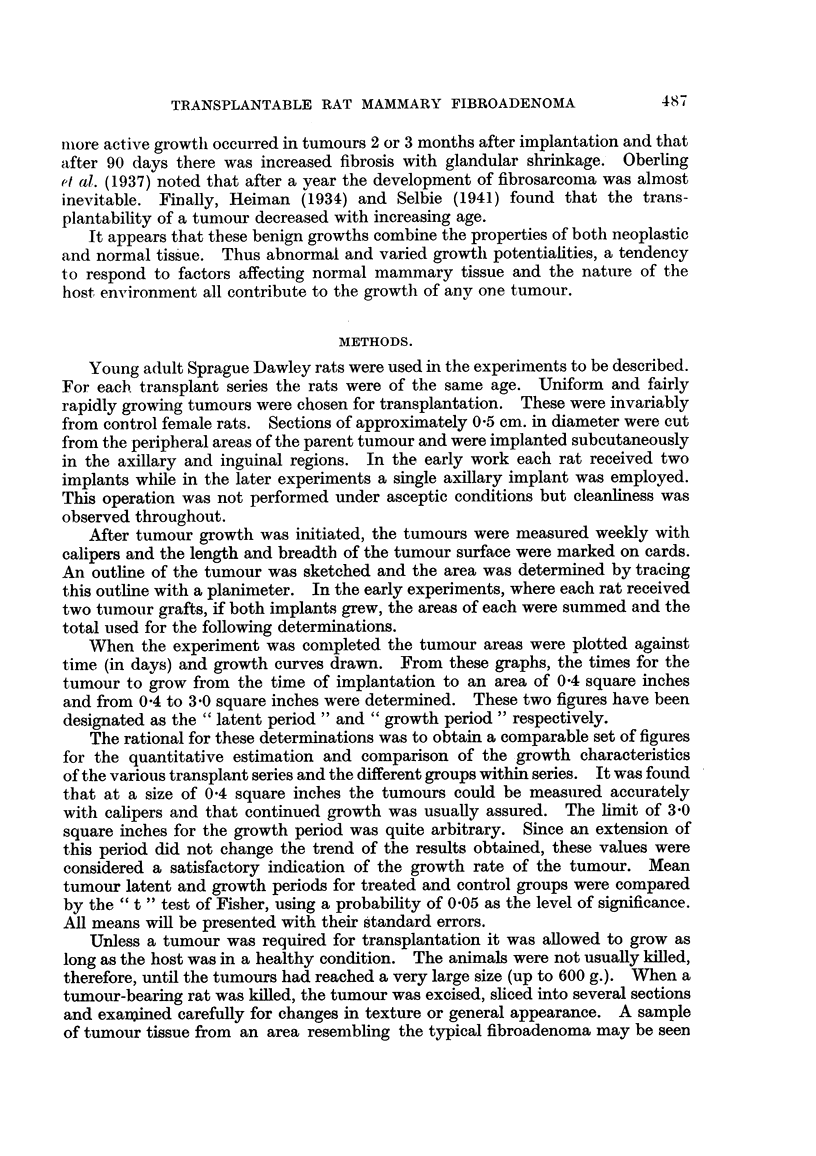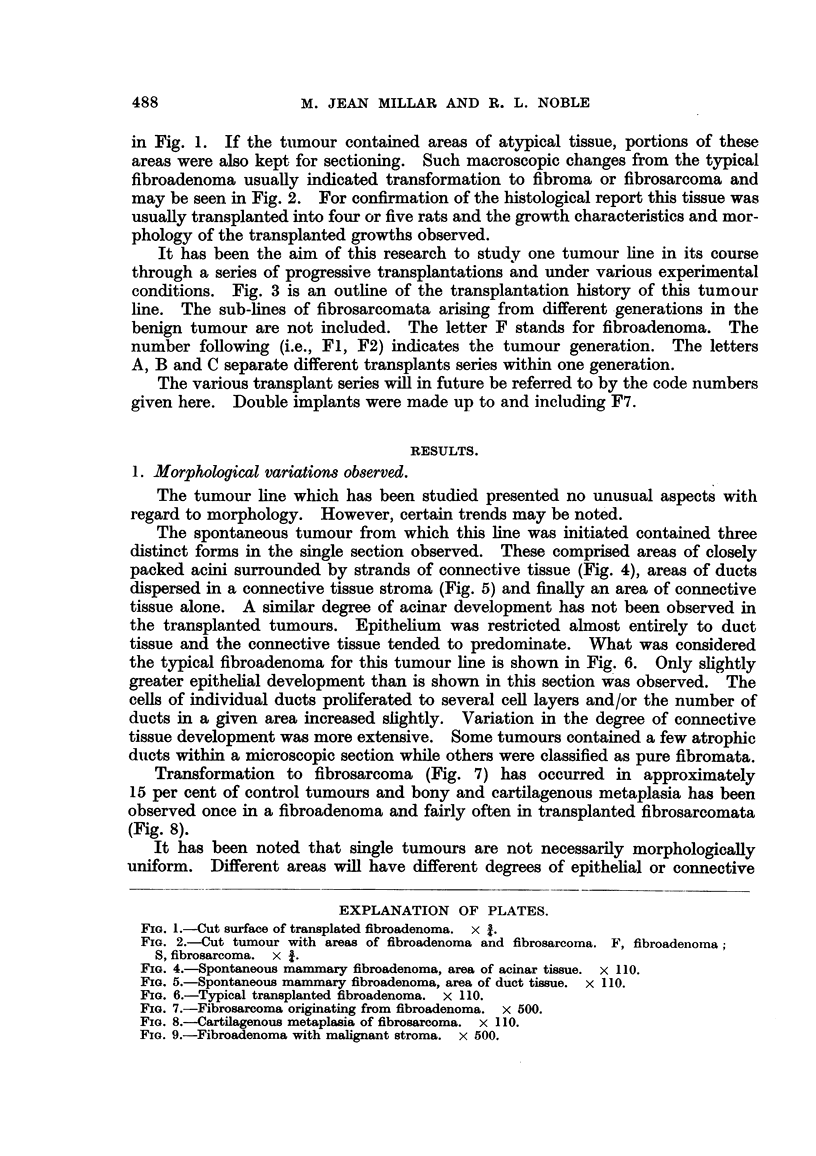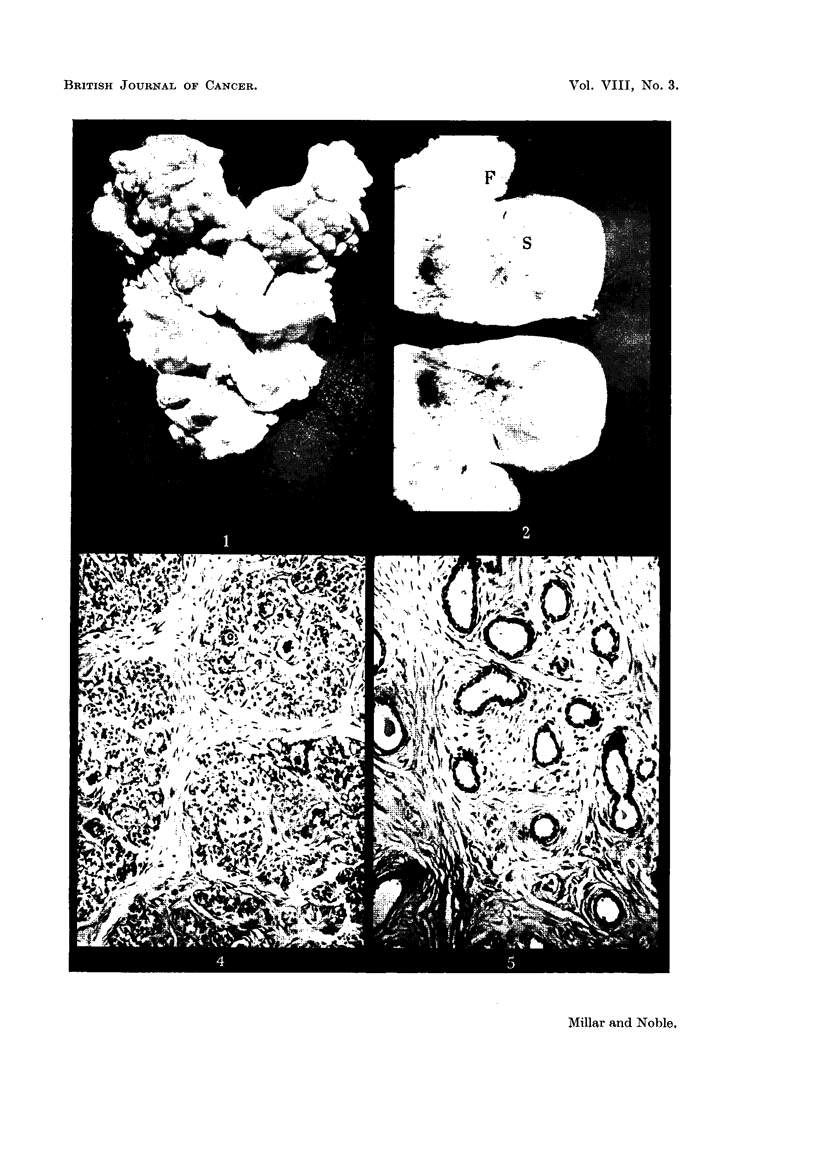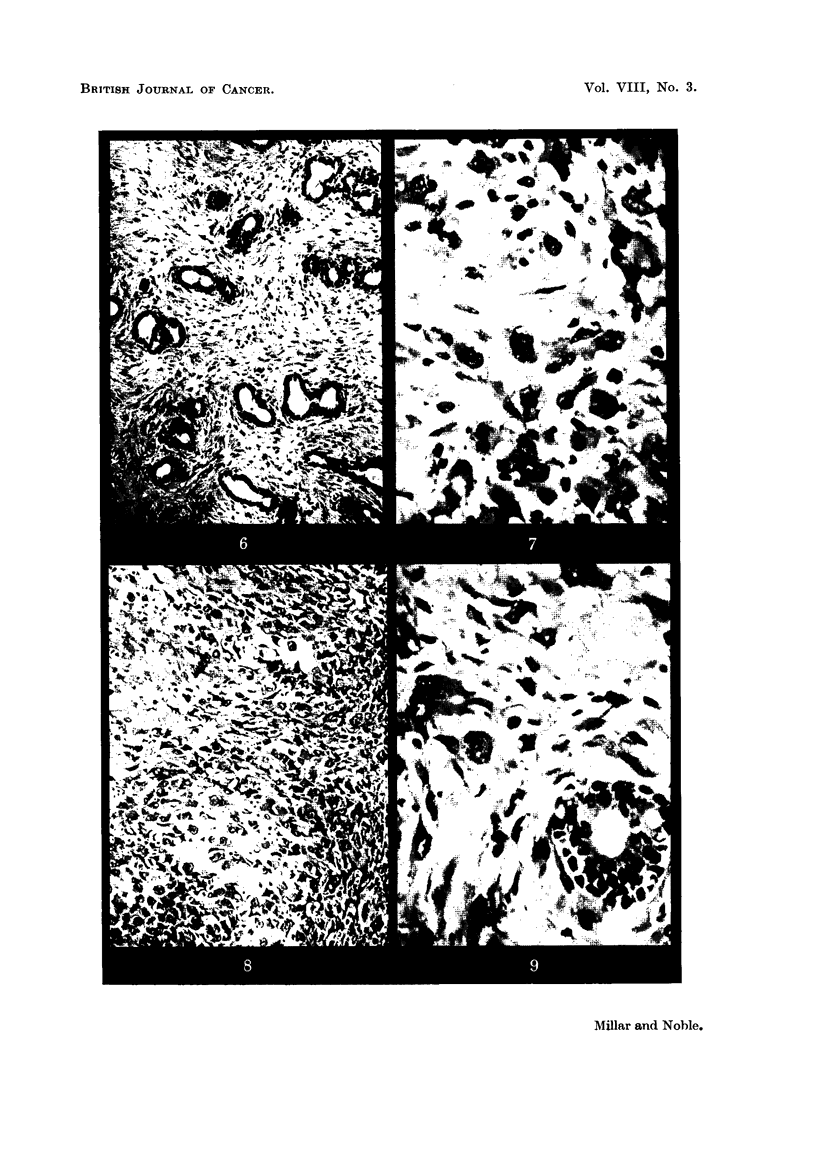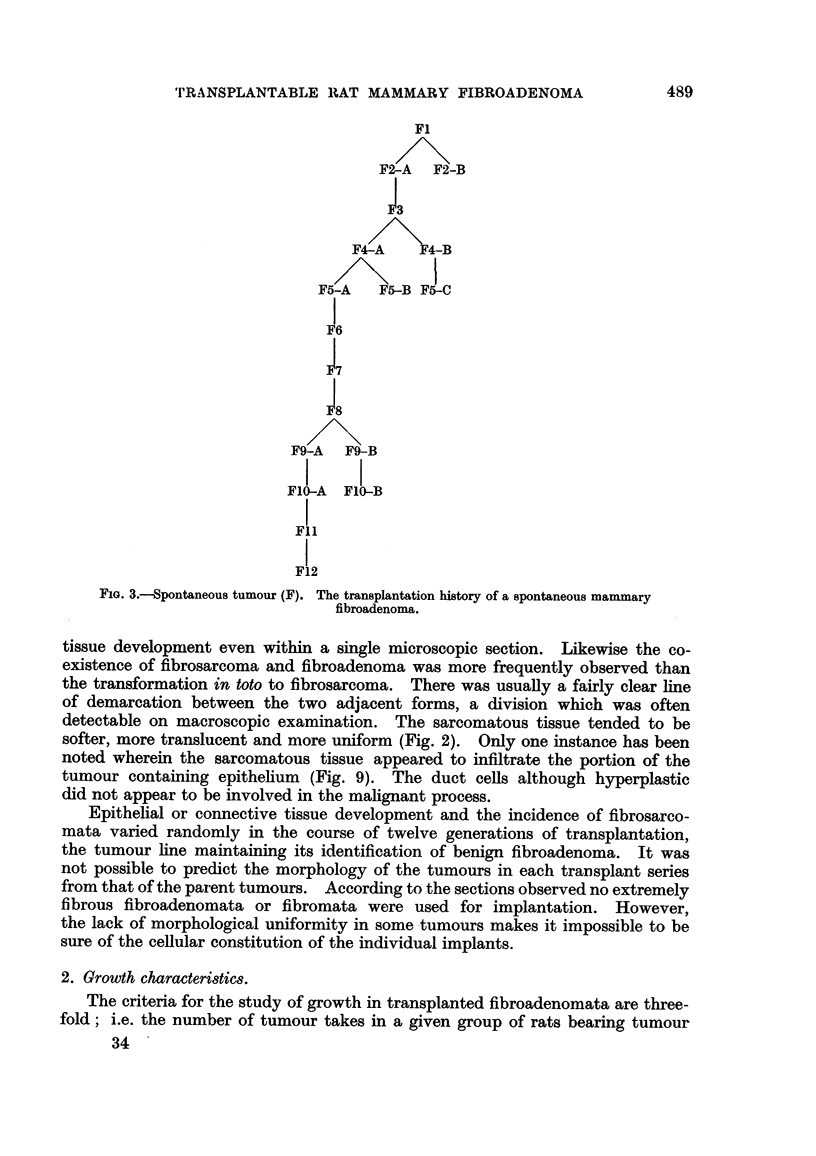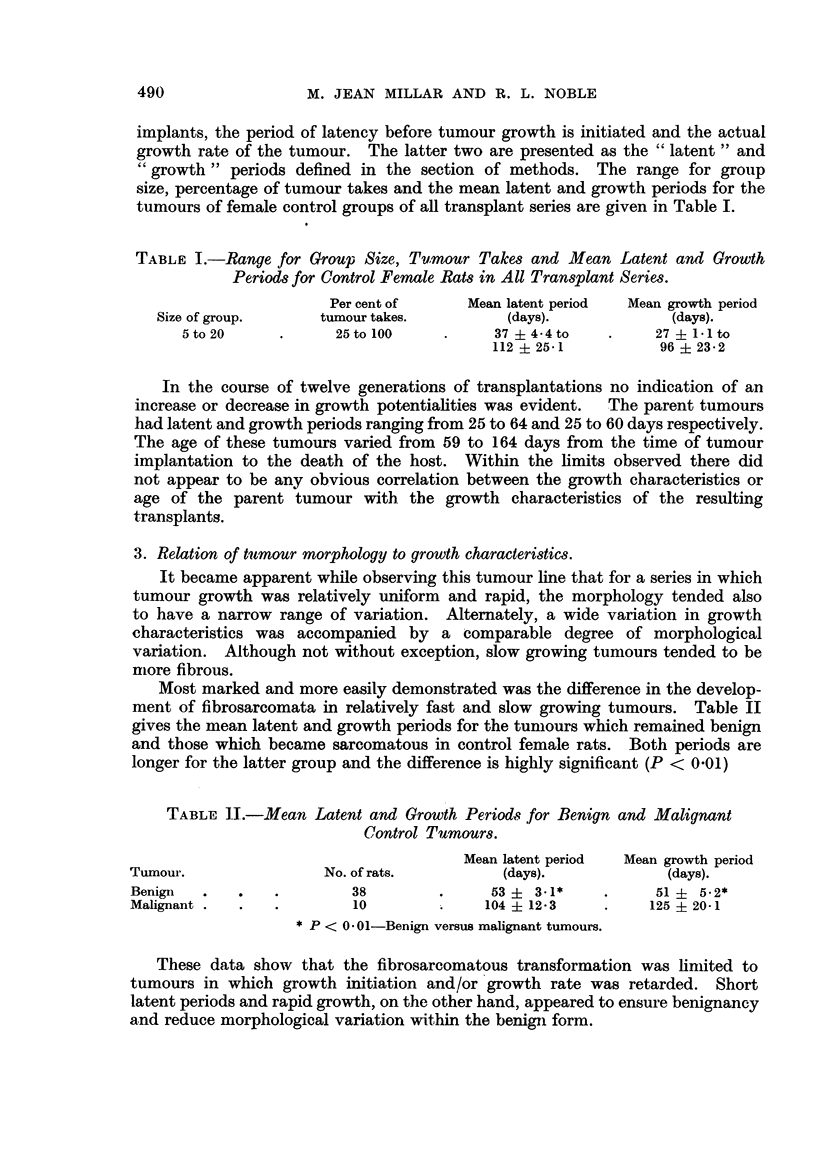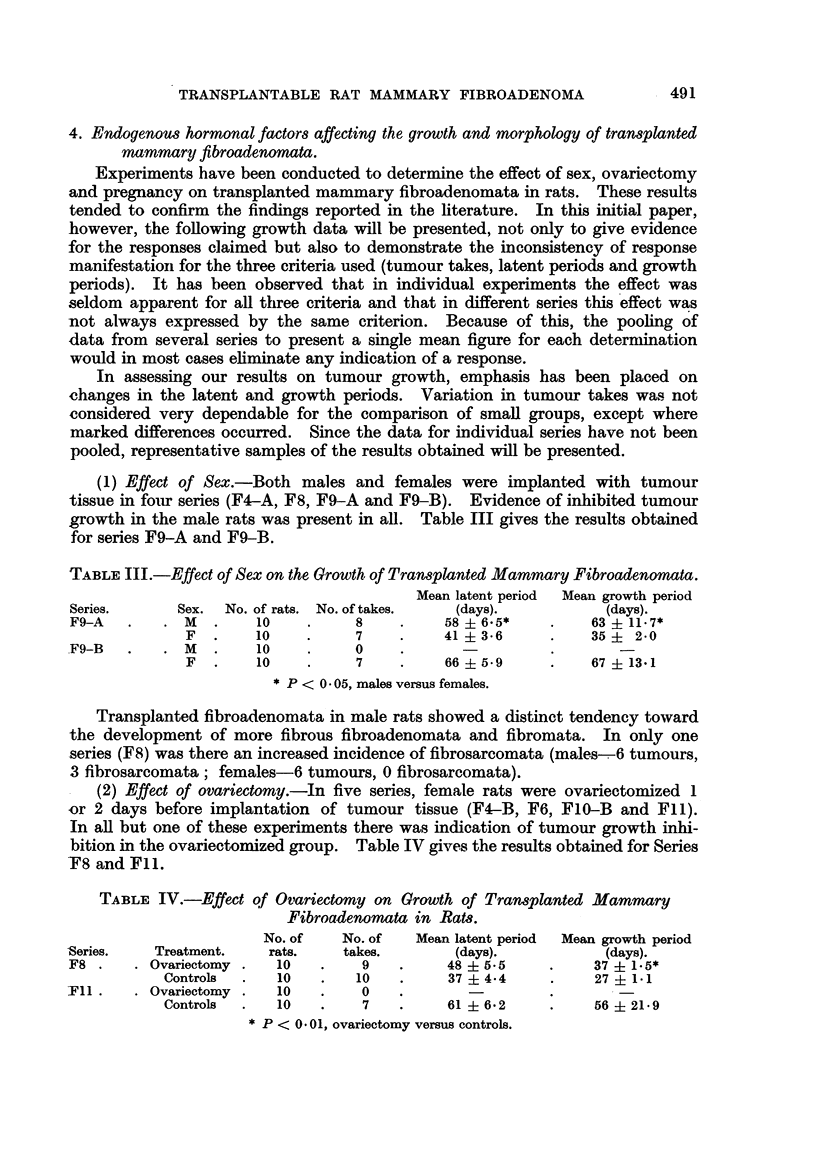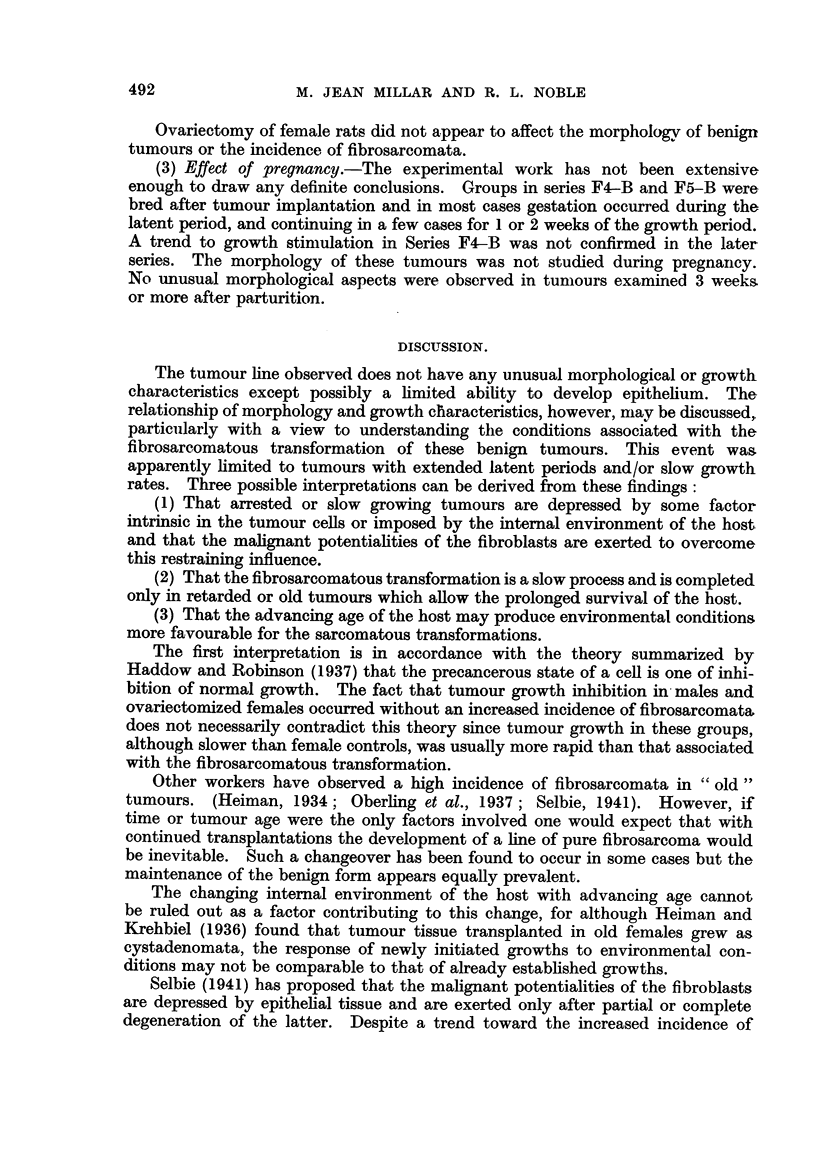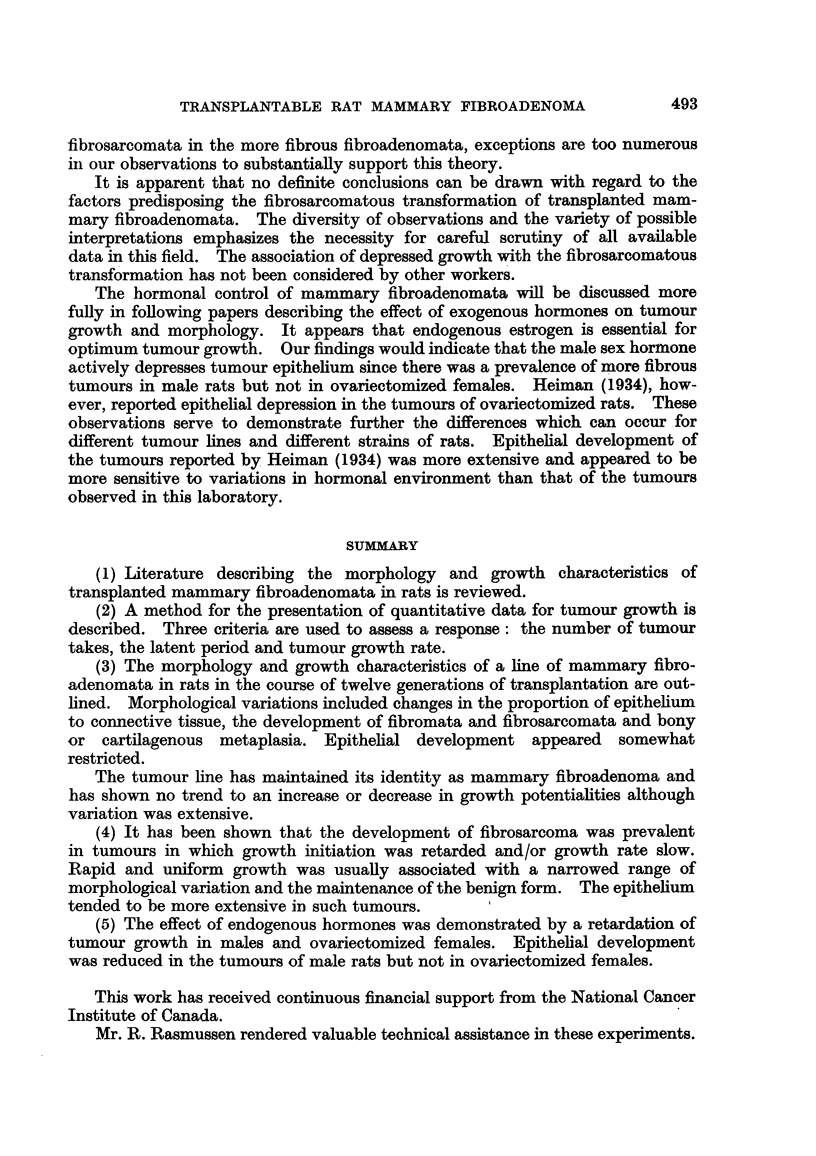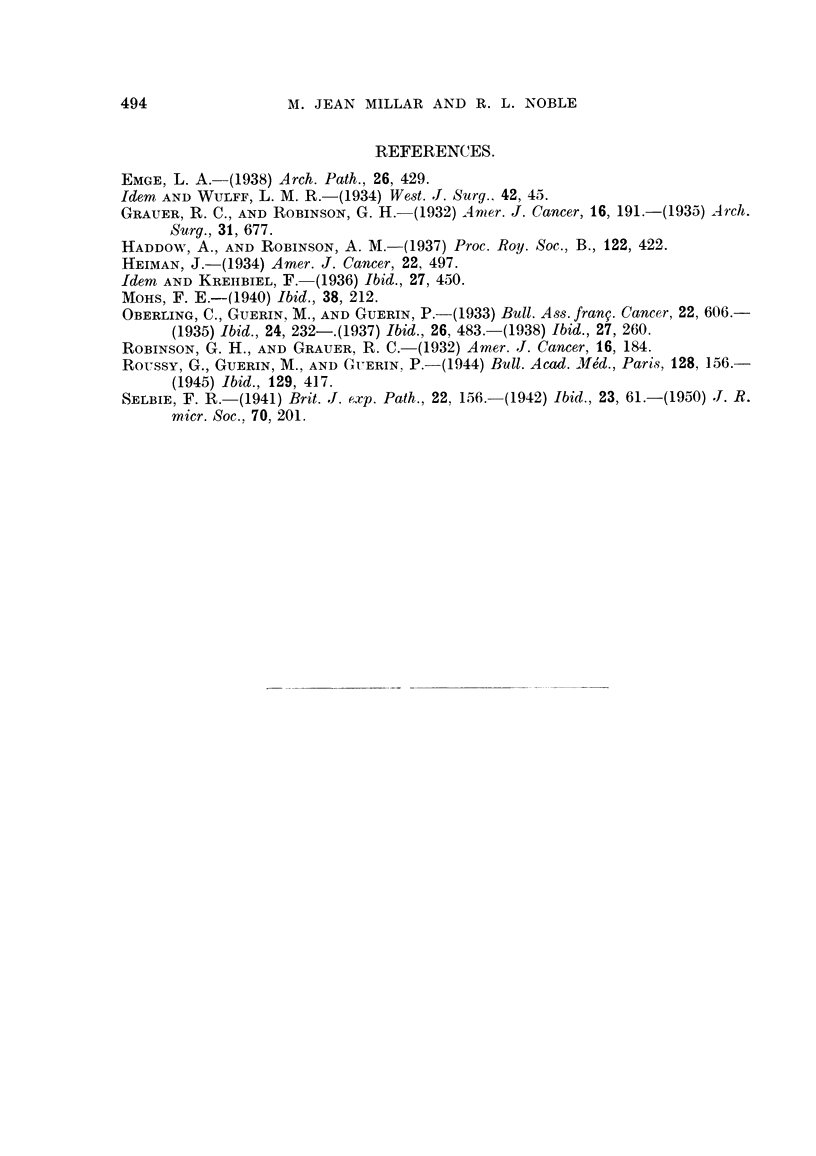# The Morphology and Growth Characteristics of a Transplantable Mammary Fibroadenoma in the Rat

**DOI:** 10.1038/bjc.1954.51

**Published:** 1954-09

**Authors:** M. Jean Millar, R. L. Noble

## Abstract

**Images:**


### THE MORPHOLOGY AND GROWTH CHARACTERISTICS OF A TRANSPLANTABLE MAMMARY FIBROADENOMA IN THE RAT.

M. JEAN MILLAR AND R. L. NOBLE.

*From the Collip Medical Research Laboratory, University of Western Ontario, London, Canada.*

Received for publication July 6, 1954.

THE successful transplantation of a spontaneous mammary fibroadenoma from a stock female rat and the realization that this type of growth presented many interesting aspects relative to both normal and neoplastic tissue led to a continuation and detailed study of this tumour line. Although the literature is fairly extensive in this field the growth potentialities of these benign tumours, as evidenced by the diversity of reported observations, was believed to justify the undertaking of such work.

In the course of experimentation three integrated but nevertheless distinct branches of research evolved. These are outlined as follows :

(1) The study of the morphology and growth characteristics of this line of mammary fibroadenoma in the course of transplantation.

(2) The determination of the effect of endogenous and exogenous hormones upon the growth characteristics and morphology of these tumours.

(3) A study of the behaviour on transplantation of fibroadenomata which had undergone a fibrosarcomatous transformation and the determination of the extent to which the change to a morphologically malignant form had influenced the response to various hormonal factors known to affect the growth of the benign fibroadenoma.

The results obtained in these studies will be presented in three publications.

The major descriptive works in this field have been presented by the following : Heiman (1934), Heiman and Krehbiel (1936), Robinson and Grauer (1932), Grauer and Robinson (1932 and 1935), Emge (1938), Mohs (1940), Selbie (1941, 1942 and 1950) ; Oberling, Guerin and Guerin (1933, 1935, 1937 and 1938) and Roussy, Guerin and Guerin (1944).

From a consideration of these papers it may be noted that spontaneous and transplanted mammary fibroadenomata in rats are well encapsulated, usually lobulated growths, and pearly white or pinkish in colour. Microscopically these tumours can present the appearance of pericanalicular or intracanalicular fibroadenoma, the former predominating in frequency. The proportion of epithelium (acini and/or ducts) to connective tissue may vary considerably, from the extremes of pure adenoma and fibroma. Carcinoma and fibrosarcoma develop from these essentially benign tumours, the fibrosarcomatous transformation being a relatively common occurrence in transplanted tumours. These fibrosarcomata have been found to bear all the characteristics of malignant growths except the ability to metastasize. Selbie (1942) alone has reported the carcinomatous transformation of transplanted mammary fibroadenomata in rats. Squamous and sebacious metaplasia of epithelial elements and bony and cartilagenous metaplasia of connective tissue elements have been observed. (Oberling *et al.*, 1935, 1938 ; Selbie, 1941 ; Roussy, Guerin and Guerin, 1945.)

It appears that the morphological character of a tumour line can follow two main paths ; one, to maintain its definition as a fibroadenoma despite the development of occasional fibrosarcomata ; the other, to a progressive loss of epithelium in the course of transplantation with the eventual development of a line of pure fibroma or fibrosarcoma.

Considerable variability in the number of takes, the latent periods and the growth rates of transplanted rat fibroadenomata has been observed by most authors. Compared to common transplanted malignant tumours the fibroadenoma has a slow growth rate and can reach a very large size with no apparent harm to the host. The number of takes in a given series varies. Latent periods range from 4 weeks to over one year. The rate of growth also contains wide extremes and is sometimes irregular.

The variable nature of both the morphology and growth characteristics of the benign mammary fibroadenoma suggests its susceptibility to the environmental conditions of the host. The mammary origin of this type of tumour would also lead one to expect some hormonal influence on its growth and morphology. This has been verified by the findings of most workers in this field, for it is generally agreed that mammary fibroadenomata are more successfully propagated in female rats than in males. Heiman and Krehbiel (1936) noted also that increased takes and accelerated tumour growth initiation and growth rate occurred in castrated males with the reverse in castrated females. Mohs (1940), however, found no differences for tumour growth in intact or castrated males although castration of females produced results similar to those of the above authors. Morphologically these tumours respond to the male environment with a depression of epithelial elements and a predominance of connective tissue. Selbie (1941) observed an increased incidence of intracanalicular fibroadenomata in male rats.

The age of the host can also affect the nature of transplanted fibroadenomata, a fact which lends further evidence to the influence of sex hormones on tumour growth. Heiman and Krehbiel (1936) found that transplanted fibroadenomata grew in young immature females as cellular fibromata, in young females of breeding age as fibroadenomata with pronounced glandular hyperplasia and in old females past litter-bearing age as cystadenomata and papillary cystadenomata. These age differences were not apparent in the male. Emge (1938) reported very few takes in rats under 60 days of age, obtaining maximum takes during the period of sexual maturity and a diminished response in old animals.

The response of the mammary fibroadenoma to pregnancy and lactation seems to parallel that of normal mammary tissue at least with regard to its morphology. Some disagreement arises, however, as to the effect of pregnancy on the rate of tumour growth. Most workers have reported growth acceleration during pregnancy but Emge and Wulff (1934), using more quantitative methods, found no increase in tumour growth rates during pregancy and contended that if anything growth retardation was the trend.

Besides the hormonal environment, the genetic background of the host influences the growth of transplanted fibroadenomata. Species specificity (Heiman, 1934) and increased growth energy in selectively inbred rats (Emge, 1938) have been noted. The age of the tumour tissue itself has also been related to the growth and transplantability of these tumours. Heiman (1934) has noted that more active growth occurred in tumours 2 or 3 months after implantation and that after 90 days there was increased fibrosis with glandular shrinkage. Oberling *et al.* (1937) noted that after a year the development of fibrosarcoma was almost inevitable. Finally, Heiman (1934) and Selbie (1941) found that the transplantability of a tumour decreased with increasing age.

It appears that these benign growths combine the properties of both neoplastic and normal tissue. Thus abnormal and varied growth potentialities, a tendency to respond to factors affecting normal mammary tissue and the nature of the host environment all contribute to the growth of any one tumour.

### METHODS.

Young adult Sprague Dawley rats were used in the experiments to be described. For each transplant series the rats were of the same age. Uniform and fairly rapidly growing tumours were chosen for transplantation. These were invariably from control female rats. Sections of approximately 0·5 cm. in diameter were cut from the peripheral areas of the parent tumour and were implanted subcutaneously in the axillary and inguinal regions. In the early work each rat received two implants while in the later experiments a single axillary implant was employed. This operation was not performed under asceptic conditions but cleanliness was observed throughout.

After tumour growth was initiated, the tumours were measured weekly with calipers and the length and breadth of the tumour surface were marked on cards. An outline of the tumour was sketched and the area was determined by tracing this outline with a planimeter. In the early experiments, where each rat received two tumour grafts, if both implants grew, the areas of each were summed and the total used for the following determinations.

When the experiment was completed the tumour areas were plotted against time (in days) and growth curves drawn. From these graphs, the times for the tumour to grow from the time of implantation to an area of 0·4 square inches and from 0·4 to 3·0 square inches were determined. These two figures have been designated as the " latent period " and " growth period " respectively.

The rational for these determinations was to obtain a comparable set of figures for the quantitative estimation and comparison of the growth characteristics of the various transplant series and the different groups within series. It was found that at a size of 0·4 square inches the tumours could be measured accurately with calipers and that continued growth was usually assured. The limit of 3·0 square inches for the growth period was quite arbitrary. Since an extension of this period did not change the trend of the results obtained, these values were considered a satisfactory indication of the growth rate of the tumour. Mean tumour latent and growth periods for treated and control groups were compared by the " t " test of Fisher, using a probability of 0·05 as the level of significance. All means will be presented with their standard errors.

Unless a tumour was required for transplantation it was allowed to grow as long as the host was in a healthy condition. The animals were not usually killed, therefore, until the tumours had reached a very large size (up to 600 g.). When a tumour-bearing rat was killed, the tumour was excised, sliced into several sections and examined carefully for changes in texture or general appearance. A sample of tumour tissue from an area resembling the typical fibroadenoma may be seen in Fig. 1. If the tumour contained areas of atypical tissue, portions of these areas were also kept for sectioning. Such macroscopic changes from the typical fibroadenoma usually indicated transformation to fibroma or fibrosarcoma and may be seen in Fig. 2. For confirmation of the histological report this tissue was usually transplanted into four or five rats and the growth characteristics and morphology of the transplanted growths observed.

It has been the aim of this research to study one tumour line in its course through a series of progressive transplantations and under various experimental conditions. Fig. 3 is an outline of the transplantation history of this tumour line. The sub-lines of fibrosarcomata arising from different generations in the benign tumour are not included. The letter F stands for fibroadenoma. The number following (i.e., F1, F2) indicates the tumour generation. The letters A, B and C separate different transplants series within one generation.

The various transplant series will in future be referred to by the code numbers given here. Double implants were made up to and including F7.

### RESULTS.

### 1. *Morphological variations observed.*

The tumour line which has been studied presented no unusual aspects with regard to morphology. However, certain trends may be noted.

The spontaneous tumour from which this line was initiated contained three distinct forms in the single section observed. These comprised areas of closely packed acini surrounded by strands of connective tissue (Fig. 4), areas of ducts dispersed in a connective tissue stroma (Fig. 5) and finally an area of connective tissue alone. A similar degree of acinar development has not been observed in the transplanted tumours. Epithelium was restricted almost entirely to duct tissue and the connective tissue tended to predominate. What was considered the typical fibroadenoma for this tumour line is shown in Fig. 6. Only slightly greater epithelial development than is shown in this section was observed. The cells of individual ducts proliferated to several cell layers and/or the number of ducts in a given area increased slightly. Variation in the degree of connective tissue development was more extensive. Some tumours contained a few atrophic ducts within a microscopic section while others were classified as pure fibromata.

Transformation to fibrosarcoma (Fig. 7) has occurred in approximately 15 per cent of control tumours and bony and cartilagenous metaplasia has been observed once in a fibroadenoma and fairly often in transplanted fibrosarcomata (Fig. 8).

It has been noted that single tumours are not necessarily morphologically uniform. Different areas will have different degrees of epithelial or connective

---

EXPLANATION OF PLATES.

FIG. 1.—Cut surface of transplated fibroadenoma. × $\frac{3}{4}$.
FIG. 2.—Cut tumour with areas of fibroadenoma and fibrosarcoma. F, fibroadenoma; S, fibrosarcoma. × $\frac{3}{4}$.
FIG. 4.—Spontaneous mammary fibroadenoma, area of acinar tissue. × 110.
FIG. 5.—Spontaneous mammary fibroadenoma, area of duct tissue. × 110.
FIG. 6.—Typical transplanted fibroadenoma. × 110.
FIG. 7.—Fibrosarcoma originating from fibroadenoma. × 500.
FIG. 8.—Cartilagenous metaplasia of fibrosarcoma. × 110.
FIG. 9.—Fibroadenoma with malignant stroma. × 500.

1

2

4

5

6

7

8

9

Millar and Noble.

FIG. 3.—Spontaneous tumour (F). The transplantation history of a spontaneous mammary fibroadenoma.

tissue development even within a single microscopic section. Likewise the co-existence of fibrosarcoma and fibroadenoma was more frequently observed than the transformation *in toto* to fibrosarcoma. There was usually a fairly clear line of demarcation between the two adjacent forms, a division which was often detectable on macroscopic examination. The sarcomatous tissue tended to be softer, more translucent and more uniform (Fig. 2). Only one instance has been noted wherein the sarcomatous tissue appeared to infiltrate the portion of the tumour containing epithelium (Fig. 9). The duct cells although hyperplastic did not appear to be involved in the malignant process.

Epithelial or connective tissue development and the incidence of fibrosarcomata varied randomly in the course of twelve generations of transplantation, the tumour line maintaining its identification of benign fibroadenoma. It was not possible to predict the morphology of the tumours in each transplant series from that of the parent tumours. According to the sections observed no extremely fibrous fibroadenomata or fibromata were used for implantation. However, the lack of morphological uniformity in some tumours makes it impossible to be sure of the cellular constitution of the individual implants.

2. *Growth characteristics.*

The criteria for the study of growth in transplanted fibroadenomata are three-fold ; i.e. the number of tumour takes in a given group of rats bearing tumour implants, the period of latency before tumour growth is initiated and the actual growth rate of the tumour. The latter two are presented as the "latent" and "growth" periods defined in the section of methods. The range for group size, percentage of tumour takes and the mean latent and growth periods for the tumours of female control groups of all transplant series are given in Table I.

TABLE I.—*Range for Group Size, Tumour Takes and Mean Latent and Growth Periods for Control Female Rats in All Transplant Series.*

| Size of group. | Per cent of tumour takes. | Mean latent period (days). | Mean growth period (days). |
|---|---|---|---|
| 5 to 20 | 25 to 100 | $37 \pm 4 \cdot 4$ to $112 \pm 25 \cdot 1$ | $27 \pm 1 \cdot 1$ to $96 \pm 23 \cdot 2$ |

In the course of twelve generations of transplantations no indication of an increase or decrease in growth potentialities was evident. The parent tumours had latent and growth periods ranging from 25 to 64 and 25 to 60 days respectively. The age of these tumours varied from 59 to 164 days from the time of tumour implantation to the death of the host. Within the limits observed there did not appear to be any obvious correlation between the growth characteristics or age of the parent tumour with the growth characteristics of the resulting transplants.

3. *Relation of tumour morphology to growth characteristics.*

It became apparent while observing this tumour line that for a series in which tumour growth was relatively uniform and rapid, the morphology tended also to have a narrow range of variation. Alternately, a wide variation in growth characteristics was accompanied by a comparable degree of morphological variation. Although not without exception, slow growing tumours tended to be more fibrous.

Most marked and more easily demonstrated was the difference in the development of fibrosarcomata in relatively fast and slow growing tumours. Table II gives the mean latent and growth periods for the tumours which remained benign and those which became sarcomatous in control female rats. Both periods are longer for the latter group and the difference is highly significant ($P < 0{\cdot}01$)

TABLE II.—*Mean Latent and Growth Periods for Benign and Malignant Control Tumours.*

| Tumour. | No. of rats. | Mean latent period (days). | Mean growth period (days). |
|---|---|---|---|
| Benign | 38 | $53 \pm 3 \cdot 1$* | $51 \pm 5 \cdot 2$* |
| Malignant | 10 | $104 \pm 12 \cdot 3$ | $125 \pm 20 \cdot 1$ |

* $P < 0 \cdot 01$—Benign versus malignant tumours.

These data show that the fibrosarcomatous transformation was limited to tumours in which growth initiation and/or growth rate was retarded. Short latent periods and rapid growth, on the other hand, appeared to ensure benignancy and reduce morphological variation within the benign form.

4. *Endogenous hormonal factors affecting the growth and morphology of transplanted mammary fibroadenomata.*

Experiments have been conducted to determine the effect of sex, ovariectomy and pregnancy on transplanted mammary fibroadenomata in rats. These results tended to confirm the findings reported in the literature. In this initial paper, however, the following growth data will be presented, not only to give evidence for the responses claimed but also to demonstrate the inconsistency of response manifestation for the three criteria used (tumour takes, latent periods and growth periods). It has been observed that in individual experiments the effect was seldom apparent for all three criteria and that in different series this effect was not always expressed by the same criterion. Because of this, the pooling of data from several series to present a single mean figure for each determination would in most cases eliminate any indication of a response.

In assessing our results on tumour growth, emphasis has been placed on changes in the latent and growth periods. Variation in tumour takes was not considered very dependable for the comparison of small groups, except where marked differences occurred. Since the data for individual series have not been pooled, representative samples of the results obtained will be presented.

(1) *Effect of Sex.*—Both males and females were implanted with tumour tissue in four series (F4–A, F8, F9–A and F9–B). Evidence of inhibited tumour growth in the male rats was present in all. Table III gives the results obtained for series F9–A and F9–B.

TABLE III.—*Effect of Sex on the Growth of Transplanted Mammary Fibroadenomata.*

| Series. | Sex. | No. of rats. | No. of takes. | Mean latent period (days). | Mean growth period (days). |
|---|---|---|---|---|---|
| F9–A | M | 10 | 8 | $58 \pm 6{\cdot}5$* | $63 \pm 11{\cdot}7$* |
| | F | 10 | 7 | $41 \pm 3{\cdot}6$ | $35 \pm 2{\cdot}0$ |
| F9–B | M | 10 | 0 | — | — |
| | F | 10 | 7 | $66 \pm 5{\cdot}9$ | $67 \pm 13{\cdot}1$ |

* $P < 0{\cdot}05$, males versus females.

Transplanted fibroadenomata in male rats showed a distinct tendency toward the development of more fibrous fibroadenomata and fibromata. In only one series (F8) was there an increased incidence of fibrosarcomata (males—6 tumours, 3 fibrosarcomata ; females—6 tumours, 0 fibrosarcomata).

(2) *Effect of ovariectomy.*—In five series, female rats were ovariectomized 1 or 2 days before implantation of tumour tissue (F4–B, F6, F10–B and F11). In all but one of these experiments there was indication of tumour growth inhibition in the ovariectomized group. Table IV gives the results obtained for Series F8 and F11.

TABLE IV.—*Effect of Ovariectomy on Growth of Transplanted Mammary Fibroadenomata in Rats.*

| Series. | Treatment. | No. of rats. | No. of takes. | Mean latent period (days). | Mean growth period (days). |
|---|---|---|---|---|---|
| F8 | Ovariectomy | 10 | 9 | $48 \pm 5{\cdot}5$ | $37 \pm 1{\cdot}5$* |
| | Controls | 10 | 10 | $37 \pm 4{\cdot}4$ | $27 \pm 1{\cdot}1$ |
| F11 | Ovariectomy | 10 | 0 | — | — |
| | Controls | 10 | 7 | $61 \pm 6{\cdot}2$ | $56 \pm 21{\cdot}9$ |

* $P < 0{\cdot}01$, ovariectomy versus controls.

Ovariectomy of female rats did not appear to affect the morphology of benign tumours or the incidence of fibrosarcomata.

(3) *Effect of pregnancy.*—The experimental work has not been extensive enough to draw any definite conclusions. Groups in series F4–B and F5–B were bred after tumour implantation and in most cases gestation occurred during the latent period, and continuing in a few cases for 1 or 2 weeks of the growth period. A trend to growth stimulation in Series F4–B was not confirmed in the later series. The morphology of these tumours was not studied during pregnancy. No unusual morphological aspects were observed in tumours examined 3 weeks or more after parturition.

### DISCUSSION.

The tumour line observed does not have any unusual morphological or growth characteristics except possibly a limited ability to develop epithelium. The relationship of morphology and growth characteristics, however, may be discussed, particularly with a view to understanding the conditions associated with the fibrosarcomatous transformation of these benign tumours. This event was apparently limited to tumours with extended latent periods and/or slow growth rates. Three possible interpretations can be derived from these findings :

(1) That arrested or slow growing tumours are depressed by some factor intrinsic in the tumour cells or imposed by the internal environment of the host and that the malignant potentialities of the fibroblasts are exerted to overcome this restraining influence.

(2) That the fibrosarcomatous transformation is a slow process and is completed only in retarded or old tumours which allow the prolonged survival of the host.

(3) That the advancing age of the host may produce environmental conditions more favourable for the sarcomatous transformations.

The first interpretation is in accordance with the theory summarized by Haddow and Robinson (1937) that the precancerous state of a cell is one of inhibition of normal growth. The fact that tumour growth inhibition in males and ovariectomized females occurred without an increased incidence of fibrosarcomata does not necessarily contradict this theory since tumour growth in these groups, although slower than female controls, was usually more rapid than that associated with the fibrosarcomatous transformation.

Other workers have observed a high incidence of fibrosarcomata in " old " tumours. (Heiman, 1934 ; Oberling *et al.*, 1937 ; Selbie, 1941). However, if time or tumour age were the only factors involved one would expect that with continued transplantations the development of a line of pure fibrosarcoma would be inevitable. Such a changeover has been found to occur in some cases but the maintenance of the benign form appears equally prevalent.

The changing internal environment of the host with advancing age cannot be ruled out as a factor contributing to this change, for although Heiman and Krehbiel (1936) found that tumour tissue transplanted in old females grew as cystadenomata, the response of newly initiated growths to environmental conditions may not be comparable to that of already established growths.

Selbie (1941) has proposed that the malignant potentialities of the fibroblasts are depressed by epithelial tissue and are exerted only after partial or complete degeneration of the latter. Despite a trend toward the increased incidence of fibrosarcomata in the more fibrous fibroadenomata, exceptions are too numerous in our observations to substantially support this theory.

It is apparent that no definite conclusions can be drawn with regard to the factors predisposing the fibrosarcomatous transformation of transplanted mammary fibroadenomata. The diversity of observations and the variety of possible interpretations emphasizes the necessity for careful scrutiny of all available data in this field. The association of depressed growth with the fibrosarcomatous transformation has not been considered by other workers.

The hormonal control of mammary fibroadenomata will be discussed more fully in following papers describing the effect of exogenous hormones on tumour growth and morphology. It appears that endogenous estrogen is essential for optimum tumour growth. Our findings would indicate that the male sex hormone actively depresses tumour epithelium since there was a prevalence of more fibrous tumours in male rats but not in ovariectomized females. Heiman (1934), however, reported epithelial depression in the tumours of ovariectomized rats. These observations serve to demonstrate further the differences which can occur for different tumour lines and different strains of rats. Epithelial development of the tumours reported by Heiman (1934) was more extensive and appeared to be more sensitive to variations in hormonal environment than that of the tumours observed in this laboratory.

### SUMMARY

(1) Literature describing the morphology and growth characteristics of transplanted mammary fibroadenomata in rats is reviewed.

(2) A method for the presentation of quantitative data for tumour growth is described. Three criteria are used to assess a response : the number of tumour takes, the latent period and tumour growth rate.

(3) The morphology and growth characteristics of a line of mammary fibroadenomata in rats in the course of twelve generations of transplantation are outlined. Morphological variations included changes in the proportion of epithelium to connective tissue, the development of fibromata and fibrosarcomata and bony or cartilagenous metaplasia. Epithelial development appeared somewhat restricted.

The tumour line has maintained its identity as mammary fibroadenoma and has shown no trend to an increase or decrease in growth potentialities although variation was extensive.

(4) It has been shown that the development of fibrosarcoma was prevalent in tumours in which growth initiation was retarded and/or growth rate slow. Rapid and uniform growth was usually associated with a narrowed range of morphological variation and the maintenance of the benign form. The epithelium tended to be more extensive in such tumours.

(5) The effect of endogenous hormones was demonstrated by a retardation of tumour growth in males and ovariectomized females. Epithelial development was reduced in the tumours of male rats but not in ovariectomized females.

This work has received continuous financial support from the National Cancer Institute of Canada.

Mr. R. Rasmussen rendered valuable technical assistance in these experiments.

### REFERENCES.

EMGE, L. A.—(1938) *Arch. Path.*, **26**, 429.
*Idem* AND WULFF, L. M. R.—(1934) *West. J. Surg.*, **42**, 45.
GRAUER, R. C., AND ROBINSON, G. H.—(1932) *Amer. J. Cancer*, **16**, 191.—(1935) *Arch. Surg.*, **31**, 677.
HADDOW, A., AND ROBINSON, A. M.—(1937) *Proc. Roy. Soc.*, B., **122**, 422.
HEIMAN, J.—(1934) *Amer. J. Cancer*, **22**, 497.
*Idem* AND KREHBIEL, F.—(1936) *Ibid.*, **27**, 450.
MOHS, F. E.—(1940) *Ibid.*, **38**, 212.
OBERLING, C., GUERIN, M., AND GUERIN, P.—(1933) *Bull. Ass. franç. Cancer*, **22**, 606.—(1935) *Ibid.*, **24**, 232—.(1937) *Ibid.*, **26**, 483.—(1938) *Ibid.*, **27**, 260.
ROBINSON, G. H., AND GRAUER, R. C.—(1932) *Amer. J. Cancer*, **16**, 184.
ROUSSY, G., GUERIN, M., AND GUERIN, P.—(1944) *Bull. Acad. Méd., Paris*, **128**, 156.—(1945) *Ibid.*, **129**, 417.
SELBIE, F. R.—(1941) *Brit. J. exp. Path.*, **22**, 156.—(1942) *Ibid.*, **23**, 61.—(1950) *J. R. micr. Soc.*, **70**, 201.